# Individualizing immunosuppression in lung transplantation

**DOI:** 10.21542/gcsp.2018.5

**Published:** 2018-03-14

**Authors:** Jennifer K. McDermott, Reda E. Girgis

**Affiliations:** 1 Richard DeVos Heart and Lung Transplant Program, Spectrum Health, Grand Rapids, Michigan; 2 Michigan State University College of Human Medicine, Grand Rapids, Michigan

## Abstract

Immunosuppression management after lung transplantation continues to evolve, with an increasing number of agents available for use in various combinations allowing for more choice and individualization of immunosuppressive therapy. Therapeutic developments have led to improved outcomes including lower acute rejection rates and improved survival. However, a one size fits all approach for any immunosuppressive strategy may not be best suited to the individual patient and ultimately patient specific factors must be considered when designing the immunosuppressive regimen. Recipient factors including age, race, co-morbidities, immunologic risk, genetic polymorphisms, concomitant and previous pharmacotherapy, and overall immunosuppression burden should be considered. There are several significant drug-drug interactions with select immunosuppressive agents utilized in lung transplant pharmacotherapy that must be considered when choosing and devising a dosing strategy for an individual immunosuppressive agent. Herein, considerations for immunosuppression management in the individual patient will be reviewed.

## Introduction

**Figure 1.**
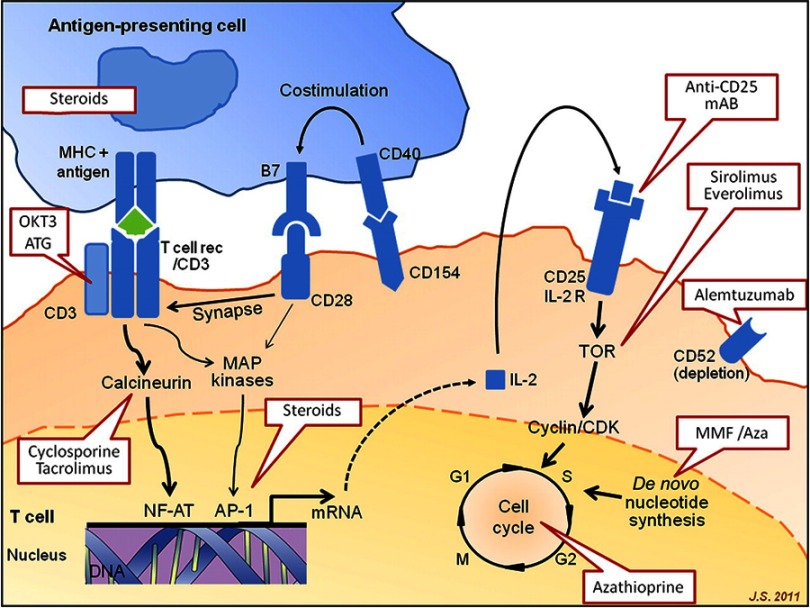
Individual immunosuppressive drugs and sites of action. Reprinted with permission from: Luis Alonso-Pulpón et al. Heart 2012;98:878-889.

Over the past thirty years, since the first successful long-term lung transplant in 1983, lung transplantation and associated immunosuppressive therapy have greatly evolved. An increasing number of induction and maintenance immunosuppressive agents have become available over time for use in various combinations allowing for more choice and personalization of immunosuppressive therapy (Figure 1, Table 1). Despite several advancements, there are pros and cons to each immunosuppressive agent and a regimen that leads to prolonged survival yet is void of associated morbidity including infection, malignancy, and drug-related toxicities has not been identified.

**Table 1:** Immunosuppressive agents.

Immunosuppressive Agent	Class	Uses in Lung Transplantation (Off-label)	Adverse Effects	Monitoring Parameters	Additional Considerations
Basiliximab (Simulect^®^)	Anti-CD25 monoclonal antibody; binds to the *α* subunit of the IL-2 receptor present only on activated and non-resting T cells	Induction	Rare; possible hypersensitivity reaction	None	
Anti-thymocyte globulin, rabbit (Thymoglobulin^^®^^)	Polyclonal antibody; polyclonal IgG against human T lymphocytes derived from rabbits; reduces the number of circulating T lymphocytes, which alters T cell activation, homing, and cytotoxic function	Induction, treatment of rejection, treatment of chronic lung allograft dysfunction	Leukopenia, thrombocytopenia, fever, chills, dyspnea, pulmonary edema, tachycardia, hypotension, phlebitis, pruritis, erythema, rash, serum sickness, infection	Vital signs, CBC, absolute lymphocyte count, CD3 count	Pre-medication recommended with diphenhydramine and acetaminophen
Alemtuzumab (Campath^^®^^)	Anti-CD52 monoclonal antibody; binds CD52 antigen on T and B cells, NK cells, and less densely on monocytes and macrophages causing cell lysis through antibody-dependent cellular cytotoxicity resulting in profound depletion of T cells and to a lesser degree B cells and monocytes	Induction, treatment of rejection, treatment of chronic lung allograft dysfunction	Leukopenia, thrombocytopenia, headache, fever, chills, dyspnea, tachycardia, hypotension, phlebitis, pruritis, erythema, rash, infection	Vital signs, CBC, absolute lymphocyte count	Pre-medication recommended with diphenhydramine and acetaminophen
Corticosteroids: Prednisone, Prednisolone, Methylprednisolone, Dexamethasone	Inhibit NF-AT thereby blocking transcription of cytokine genes (interleukins 1, 2, 3, 5, TNF-alpha, and interferon gamma) and inhibiting cytokine production by T cells and macrophages	Induction, treatment of rejection, maintenance	Hyperglycemia, hypertension, hyperlipidemia, psychosis, mood swings, insomnia, photosensitivity, acne, osteoporosis, bone fractures, avascular necrosis, weight gain, fluid retention, increased appetite, hirsutism, Cushing’s syndrome, menstrual irregularities, growth retardation, GI disturbance, cataracts, impaired wound healing, infection	Glucose, blood pressure, fasting lipid panel, weight, DEXA scan, eye exams	
Tacrolimus (Prograf^^®^^, Envarsus XR^^®^^, Astagraf^^®^^)	Calcineurin inhibitor; results in blockade of signal transduction by NF-AT, thereby preventing gene transcription for formation of lymphokines and ultimately inhibiting T cell activation	Maintenance	Nephrotoxicity, neurotoxicity (Tac>CsA), hyperglycemia (Tac>CsA), hypertension (CsA>Tac) hyperlipidemia (CsA>Tac), hyperkalemia, hypomagnesemia, hyperuricemia, HUS/TMA, infection,gingival hyperplasia (CsA only), hirsutism (CsA only), alopecia (Tac only)	12-hour trough levels (Prograf^^®^^) or 24-hour trough levels (Envarsus XR^^®^^, Astagraf^^®^^), serum creatinine, potassium, magnesium, uric acid	
Cyclosporine (Neoral^^®^^, Gengraf^^®^^, Sandimmune^^®^^)	Calcineurin inhibitor; results in blockade of signal transduction by NF-AT, thereby preventing gene transcription for formation of lymphokines and ultimately inhibiting T cell activation	Maintenance		12-hour trough levels or 2-hour post-dose levels, serum creatinine, potassium, magnesium, uric acid	Modified formulations (Neoral^^®^^, Gengraf^^®^^) are not bioequivalent to non-modified formulations (Sandimmune^^®^^)
Mycophenolate mofetil (Cellcept^^®^^): Pro-drug of mycophenolic acid	Anti-metabolite/cell cycle inhibitor; inhibits lymphocyte purine synthesis by reversibly and noncompetitively inhibiting IMPDH	Maintenance	Nausea, vomiting, diarrhea, abdominal pain, leukopenia, thrombocytopenia, anemia, infection, cytomegalovirus infection	CBC, pregnancy test in women of childbearing potential (REMS)	REMS requirements to communicate increased risks of pregnancy loss and congenital malformations associated with mycophenolate exposure during pregnancy. Females of reproductive potential must be counseled on pregnancy prevention and planning and need to report pregnancies to the Mycophenolate Pregnancy Registry.
Mycophenolate sodium (Myfortic^^®^^): Delayed-release, enteric coated tablet of mycophenolic acid					
Azathioprine (Imuran^^®^^)	Anti-metabolite/cell cycle inhibitor; Metabolized to 6-mercaptopurine which is incorporated into nucleic acids (substitutes for the purine base guanine) ultimately inhibiting DNA and RNA synthesis	Maintenance	Leukopenia, thrombocytopenia, macrocytic anemia, nausea, vomiting, abdominal pain, alopecia, pancreatitis, hepatotoxicity, infection	CBC, LFT, amylase, lipase, TPMT enzyme level	Low or absent TPMT activity is associated with increased azathioprine associated myelosuppression
Sirolimus (Rapamune^^®^^)	m-TOR inhibitor/proliferation signal inhibitor; blocks signal transduction pathways ultimately inhibiting IL-2 and other cytokine induced activation and proliferation of T and B cells	Maintenance	Thrombocytopenia, leukopenia, anemia, hyperlipidemia, impaired wound healing, wound related reactions, peripheral edema, mouth ulcers, bone pain, diarrhea, proteinuria, pneumonitis, pneumonia, venous thromboembolism, HUS/TMA, infection	24-hour trough levels (C0), fasting lipid panel, CBC, LFT	Frequent dosage adjustments based on non-steady state sirolimus concentrations can lead to overdosing or underdosing due to the long elimination half-life of sirolimus
Everolimus (Zortress^^®^^)	m-TOR inhibitor/proliferation signal inhibitor; blocks signal transduction pathways ultimately inhibiting IL-2 and other cytokine induced activation and proliferation of T and B cells	Maintenance	Thrombocytopenia, leukopenia, anemia, hyperlipidemia, impaired wound healing, wound related reactions, peripheral edema, mouth ulcers, bone pain, diarrhea, proteinuria, pneumonitis, pneumonia, venous thromboembolism, HUS/TMA, infection	12-hour trough levels (C0), fasting lipid panel, CBC, LFT	
Belatacept (Nulojix^^®^^)	Co-stimulation blocker; blocks the CD28 mediated costimulation of T lymphocytes by binding to CD80 and CD86 on antigen-presenting cells	Maintenance	Fever, hypertension, headache, cough, anemia, leukopenia, nausea, vomiting, diarrhea, constipation, peripheral edema, PTLD, PML, infection	EBV serostatus (prior to treatment)	Contraindicated in transplant recipients who are EBV seronegative or with unknown EBV serostatus

**Notes.**

Abbreviations CBC complete blood count CsA cyclosporine DEXA dual-energy x-ray absorptiometry EBV Epstein-Barr virus HUS/TMA hemolytic uremic syndrome/thrombotic microangiopathy IMPDH inosine monophosphate dehydrogenase LFT liver function tests m-TOR mammalian target of rapamycin NF-AT nuclear factor of activated T-cells PML progressive multifocal leukoencephalopathy PTLD post-transplant lymphoproliferative disorder REMS risk evaluation and mitigation strategy Tac tacrolimus TPMT thiopurine methyltransferase

Calcineurin inhibitors (CNI) have reduced rejection rates and improved overall survival, but are associated with significant adverse effects including nephrotoxicity. Chronic kidney disease remains one of the most common complications in lung transplant patients at 5 years post-transplant; 14% have creatinine >2.5 mg/dL, 2.4% are on maintenance dialysis, and 0.9% require kidney transplant^1^. While the majority of lung transplant recipients continue to be maintained on a CNI, studies have been conducted aiming to minimize these agents given their adverse effect profiles. Corticosteroids continue to be a cornerstone of immunosuppressive therapy and are utilized as part of the maintenance immunosuppressive regimen in over 90 percent of lung transplant recipients^1–3^, but because they also have multiple side effects including hypertension, glucose intolerance, hyperlipidemia, weight gain, and osteoporosis, strategies have been employed to minimize dosing over time. Cell cycle inhibitors are frequently utilized but can cause gastrointestinal and hematologic adverse effects leading to need for dose adjustment or alternative therapy. mTOR inhibitors have been utilized for various reasons after lung transplantation including as a cell cycle inhibitor alternative, to minimize CNI dosing, as an adjunct agent for rejection, or in patients that develop malignancy, but high rates of adverse events leading to discontinuation have limited their use.

When designing or adjusting an immunosuppressive regimen, recipient factors including age, race, co-morbidities, immunologic risk, genetic polymorphisms, concomitant and previous pharmacotherapy, and overall immunosuppression burden should be considered. There are several significant drug-drug interactions with select immunosuppressive agents utilized in lung transplant pharmacotherapy that must be considered when choosing and devising a dosing strategy for an individual immunosuppressive agent (Table 2). Herein, considerations for immunosuppression management in the individual patient will be reviewed.

**Table 2:** Select cytochrome P450 3A interactions that affect tacrolimus, cyclosporine, sirolimus and everolimus^63,80^^*^.

CYP 450 3A Inhibitors	CYP 450 3A Inducers
Clotrimazole, fluconazole, ketoconazole, itraconazole, posaconazole, voriconazole, isavuconazole	Phenytoin, fosphenytoin, carbamazepine, oxcarbazepine, phenobarbital
Erythromycin, clarithromycin	Rifampin, rifabutin, rifapentine
Diltiazem, verapamil	Bosentan
Ritonavir, darunavir, atazanavir, lopinavir, saquinavir, indinavir, nelfinavir, fosamprenavir, tipranavir	Modafinil
Cobicistat	Efavirenz, nevirapine, etravirine
Telaprevir, boceprevir, grazoprevir	Nafcillin
Conivaptan	St. John’s wort
Nefazodone	
Aprepitant	
Isoniazid	
Amiodarone, dronedarone	
Cimetidine	
Grapefruit, star fruit, pomegranate, ginkgo biloba	

**Notes.**

* Note that this is not an exhaustive list.

## Induction immunosuppression

Induction immunosuppression is intense prophylactic therapy including a potent targeted agent utilized at the time of the transplant to prevent early acute rejection. Three specialized induction agents are currently utilized in lung transplantation; a non-lymphocyte depleting agent: basiliximab (Simulect^®^) and two T-cell depleting agents: rabbit anti-thymocyte globulin (rATG, Thymoglobulin^®^) and alemtuzumab (Campath^®^), Figure 1, Table 1. Basiliximab is a monoclonal antibody directed against CD25, the interleukin-2 receptor alpha chain of activated T cells, and is well tolerated with minimal adverse effects^4^. rATG is prepared by immunizing rabbits with human thymocytes with resulting rabbit immune globulins against human T-cells. rATG reduces the number of circulating T-lymphocytes, which alters T-cell activation, homing, and cytotoxic function and ultimately affects cell-mediated and humoral immunity^5–7^. Alemtuzumab is a humanized monoclonal antibody targeting CD52 which is located on T and B cells, NK cells, and to a lesser degree monocytes and macrophages^8,9^. The resultant antibody-dependent cellular cytotoxicity results in profound depletion of T cells and to a lesser degree B cells and monocytes. Adverse effects of both rATG and alemtuzumab include myelosuppression (leukopenia and thrombocytopenia) as well as infusion related reactions due to cytokine release.

Over the past 20 years, use of these agents in lung transplantation has significantly increased. In the International Society for Heart and Lung Transplantation (ISHLT) registry data from 2014, 69% of patients received induction immunosuppression. Most patients received an interleukin-2 receptor antagonist (IL-2 RA) (57%) whereas less received T cell depleting therapy: alemtuzumab (8%) and anti-thymocyte globulin (5%)^3^. Registry data suggest that induction therapy is associated with a reduction in early acute rejection, as well as improved survival and freedom from bronchiolitis obliterans syndrome (BOS). Furuya et al. reviewed the United Network for Organ Sharing (UNOS) database to examine the effect of induction therapy for adult bilateral lung transplant recipients. Median survival was longer for alemtuzumab and basiliximab recipients compared with patients who received no induction (2321 versus 2352 versus 1967 days, *p* = 0.001) and both agents were independently associated with survival. In addition, alemtuzumab treated recipients had a lower incidence of BOS at 5 years (22.7% versus 55.4 versus 55.9%)^10^. ISHLT registry data from July 2004 through June 2015 demonstrates a lower proportion of IL-2RA treated recipients experiencing rejection through one year as compared with no induction (29.1% vs 34.2%, *p* < 0.05). Unexpectedly, patients receiving polyclonal anti-thymocyte globulin and alemtuzumab experienced similar rejection rates compared with no induction (31.6% vs 36% vs 34.2% *p* > 0.05)^3^. rATG compared to no induction in a single center, randomized trial of 44 lung transplant patients was associated with less early rejection (5% vs 41%; *p* = 0.01), although overall rejection incidence did not differ (rATG: 62%; control: 68%; *p* = 0.52). The incidence of infections was not different between groups, but patients that received rATG had more malignancies^11^.

In contrast, two retrospective comparative analyses demonstrated that T cell depleting agents were associated with less rejection as compared with basiliximab. In a retrospective cohort study in primary lung transplant recipients, alemtuzumab was associated with significantly less grade 2 or higher rejection as compared to basiliximab at 6 months^12^. Hachem et al. found a lower incidence of acute rejection and BOS with anti-thymocyte globulin induction compared with basiliximab^13^.

One single center, prospective randomized trial of sixty lung transplant patients, compared T cell depleting agents and found alemtuzumab induction in combination with reduced dose triple maintenance immunosuppression was associated with complete absence of acute cellular rejection episodes ≥ A2 within the first year and this was significantly less than anti-thymocyte globulin plus standard dose triple maintenance immunosuppression. Survival, infections rates, renal function and incidence of malignancies did not differ between groups. Leukopenia did occur more frequently in the alemtuzumab group (76.7% vs 46.7%, *p* = 0.01)^14^.

There are no randomized controlled trials in lung transplantation comparing all three induction agents. However, in the INTAC trial conducted in kidney transplant recipients, acute rejection at three years was lower with alemtuzumab than with basiliximab in low immunological risk patients (10% versus 22%; *p* = 0.003), but among high immunological risk patients, no significant difference was observed between alemtuzumab and rATG (18% versus 15%; *p* = 0.63). T cell depleting induction agents were associated with more infections. The rate of all infectious adverse events was higher with rATG than with alemtuzumab (81% versus 60%; *p* = 0.009) and the rate of serious infectious adverse events was higher with alemtuzumab than with basiliximab (35% versus 22%; *p* = 0.02). Three year graft and patient survival did not significantly differ between groups^15^.

There is both potential concern and conflicting data regarding malignancy risk with use of T cell depleting induction agents. In kidney transplant recipients, while rATG was associated with significantly increased post-transplant lymphoproliferative disorder (PTLD) risk (*p* = 0.0025), alemtuzumab and basiliximab were not^16^. In an additional study including a total of 111,857 kidney recipients, linked transplantation and cancer registry data was examined. Alemtuzumab was associated with increased risk of non-Hodgkins lymphoma, colorectal cancer, and thyroid cancer. Polyclonal induction was associated with increased risk of melanoma^17^. Since malignancy is one of the most common complications of lung transplant occurring in 22.9% of patients at 5 years, this is an important consideration^1^.

**Figure 2.**
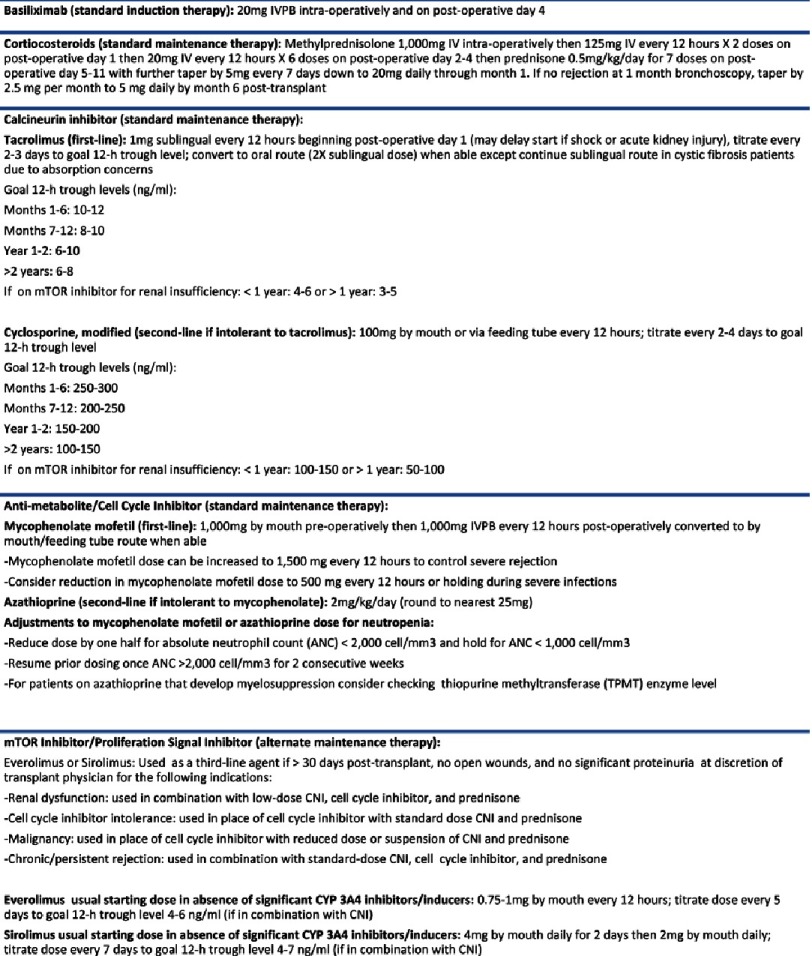
Spectrum Health Lung Transplant Immunosuppression Guideline.

Overall, induction agents tend to be associated with less rejection as compared to no induction. Infection and malignancy may be increased with the use of T cell depleting agents. At Spectrum Health, a more conservative induction immunosuppression regimen approach has been taken utilizing basiliximab (Figure 2). rATG use is reserved only for treatment of persistent or recurrent acute rejection grade A2 or greater that is refractory to high dose corticosteroids. Overall, when selecting an induction immunosuppressive agent, one must carefully consider several factors including immunological risk of the patient, cumulative immunosuppression burden, concomitant maintenance immunosuppression as well as additional patient factors including age and co-morbidities such as cardiovascular disease, infectious disease history and prior malignancy. The individual patient’s risk of rejection should be carefully weighed against potential complications due to over-immunosuppression and/or drug related toxicities.

## Maintenance immunosuppression

Triple drug maintenance immunosuppression therapy is considered standard of care after lung transplantation and consists of a CNI, tacrolimus or cyclosporine; a cell cycle inhibitor, mycophenolate or azathioprine; and a corticosteroid, prednisone. According to the ISHLT registry data, the most common regimen both at 1 and 5 years follow-up is tacrolimus, mycophenolate, and prednisone (56.2% at year 1 and 43.4% at year 5). At Spectrum Health, although first-line standard maintenance immunosuppression consists of tacrolimus, MMF, and prednisone (Figure 2), it is not uncommon for patients to require switch to alternative immunosuppressive regimens based on individual tolerability.

### Calcineurin inhibitors: Tacrolimus and cyclosporine

CNIs, first cyclosporine and later tacrolimus, are standard of care for immunosuppression after lung transplantation as they have reduced rejection rates and improved overall graft survival. Cyclosporine binds to cyclophilin whereas tacrolimus binds to FKBP-12 (Figure 1). Both inhibit the phosphatase activity of calcineurin, which regulates nuclear translocation and subsequent activation of nuclear factor of activated T-cells (NF-AT) transcription factors ultimately inhibiting T cell activation. Tacrolimus is utilized more commonly after lung transplant due to decreased risk of BOS and reports of less rejection as well as control of persistent rejection^18,19^. According to ISHLT registry data for 2015, at one year follow-up post-transplant, 93% of patients were maintained on tacrolimus^3^. Any rejection through 1 year occurred significantly less with tacrolimus as compared to cyclosporine in patients receiving concomitant mycophenolate and prednisone (28.1% vs. 37.5%, *p* < 0.05)^3^.

Hachem et al. conducted a randomized controlled trial of tacrolimus versus cyclosporine and found that lung transplant patients randomized to cyclosporine experienced the primary composite endpoint of acute rejection A score of 3 or higher, lymphocytic bronchitis B score of 4 or higher, or onset of BOS, significantly more often^20^. An additional multi-center, prospective, randomized trial found tacrolimus use was associated with a significantly reduced risk for BOS at 3 years (11.6% for tacrolimus vs 21.3% for cyclosporine, *p* = 0.037) although acute rejection and survival were similar between groups^21^. Tacrolimus may also be preferential in patients with ongoing rejection. A prospective, two-center, randomized trial comparing cyclosporine and tacrolimus found no significant difference in survival or acute rejection although 11% of cyclosporine treated patients required switch to tacrolimus to control ongoing rejection^22^. In a prospective, randomized trial of 133 lung transplant recipients receiving steroids, azathioprine and either cyclosporine (*n* = 67) or tacrolimus (*n* = 66), survival was similar between groups although there was less BOS (21.7% vs 38%, *p* = 0.025) and a trend toward less acute rejection (*p* = 0.07) with tacrolimus. Significantly more cyclosporine-treated patients required switch to tacrolimus than tacrolimus-treated patients to cyclosporine (*n* = 13 vs *n* = 2, *p* = 0.02) and the switch to tacrolimus controlled persistent acute rejection in 6 of 9 patients. The overall incidence of infections was similar, although bacterial infections were more frequent with cyclosporine (*p* = 0.0375), whereas fungal infections were more common with tacrolimus (*p* < 0.05)^23^.

Given these data and clinical experience, cyclosporine is generally now utilized mostly as a second-line agent in the setting of tolerability issues with tacrolimus as their adverse effect profiles differ (Table 1). A particular beneficial area of cyclosporine use is in the setting of tacrolimus associated neurologic adverse effects^24^.

Tacrolimus is available in several formulations including an intravenous form which is administered as a 24 h continuous infusion, oral dosage forms including extended release tablets and capsules with doses administered every 24 h as well as immediate release capsules with a usual frequency of every 12 h. In patients unable to take medications orally as well as for absorption concerns, the immediate release capsules can also be utilized for sublingual administration by opening the capsules and placing the contents under the tongue^25,26^.

At Spectrum Health, sublingual administration is frequently utilized in all patients that are not able to take medications orally. In addition, given that malabsorption problems are common in patients with cystic fibrosis; sublingual administration is often continued long-term in these patients as more consistent trough levels have been observed. A compounded suspension is also available and may be useful in pediatric patients, those unable to swallow capsules, and for administration via feeding tube. After tacrolimus feeding tube administration, care should be taken to flush and clamp for 30 to 60 minutes to allow for absorption. Bioavailability varies depending on administration route and dosage form; therefore doses may require adjustment when switching between products. When changing from the oral immediate release capsules to the intravenous form, one-fifth of the total daily oral dose is given as a continuous intravenous infusion over 24 h^27^. Immediate release capsules administered sublingually at approximately half of the oral dose achieves similar blood concentrations^25^. Conversion from immediate release tacrolimus to the extended release product Astagraf XL should be done utilizing a 1:1 ratio (mg:mg) using previously established total daily dose of immediate release then administering once daily, whereas conversion to the extended release product Envarsus XR, requires a reduction in the once-daily dose that is 80% of the total daily dose of the immediate release tacrolimus^28^. Conversion from immediate release to the compounded suspension should be done utilizing a 1:1 ratio (mg:mg).

Cyclosporine is available in an intravenous form which is given as a 24 h continuous; and both modified and non-modified oral capsules and oral solutions with a usual frequency of every 12 h. Modified and non-modified formulations are not equivalent and thus not interchangeable. Non-modified formulations have both erratic absorption and decreased bioavailability as compared to modified formulations. The modified microemulsion formulation has allowed for improved and more consistent absorption thus is preferentially utilized at our transplant center given this. Bioavailability varies depending on administration route and dosage form therefore doses may require adjustment when switching between products. When changing from the oral capsules or solution to the intravenous form, one-third of the total daily oral dose is given as a continuous intravenous infusion over 24 h^27^. Conversion from the modified capsules to the modified oral solution should be done utilizing a 1:1 ratio (mg:mg). In patients unable to take medications by mouth, the oral solution can be given via a feeding tube if needed.

Regardless of CNI dosage form, therapeutic drug monitoring with trough blood levels is routinely utilized and important to ensure efficacy and minimize adverse effects. Target trough levels are higher early post-transplant and generally decrease over time (Figure 2), but can be increased in the setting of rejection or reduced in the setting of infection, adverse effects, malignancy, and renal dysfunction to minimize nephrotoxicity. Trough blood levels should be monitored at least one to three times weekly in the immediate post-transplant period with more frequent monitoring warranted in the setting of hepatic dysfunction, gastrointestinal dysfunction (malabsorption, diarrhea, etc.), change in formulation or route (including change from brand to generic, generic to brand, or from one generic to another), and drug-drug interactions (Table 2). Both hepatic dysfunction and diarrhea can result in increased tacrolimus blood levels and resulting toxicity^29^.

### Anti-metabolite/cell cycle inhibitors: Mycophenolate and azathioprine

Mycophenolate is a potent, selective, reversible inhibitor of inosine monophosphate dehydrogenase (IMPDH), and inhibits the de novo pathway of guanosine nucleotide synthesis without incorporation into DNA. Mycophenolate has cytostatic effects on T- and B-lymphocytes since they are critically dependent on de novo synthesis of purines for their proliferation. In addition, mycophenolate has also been shown to suppress antibody formation by B-lymphocytes. Azathioprine is a pro-drug of 6-mercaptopurine and incorporates into nucleic acids (substitutes for the purine base guanine) ultimately inhibiting DNA and RNA synthesis.

Over the past 12 years, mycophenolate use has increased and azathioprine use has decreased. Per ISHLT registry data in 2015, 79 percent of patients were maintained on mycophenolate at one year follow-up^3^. Comparative data regarding outcomes including acute rejection and BOS have variable results although there are some reports of less acute rejection with mycophenolate. In a two center, non-randomized cohort study, Ross et al., found patients treated with mycophenolate mofetil (MMF) experienced significantly fewer episodes of acute rejection as compared to azathioprine treated patients (0.26 + ∕ − 0.34 vs 0.72 + ∕ − 0.43 episodes/100 patient-days, *p* < 0.01) but no significant difference in BOS (MMF: 18% vs AZA 36%, *p* = NS) at 12 months^30^. In a non-randomized single center experience of lung transplant patients treated with either MMF (*n* = 108) or azathioprine ( *n* = 48), patients treated with MMF had significantly fewer acute (*p* < 0.001), recurrent (*p* < 0.001), and less severe rejection episodes (*p* = 0.01), as well as a trend towards improved survival (*p* = 0.062), and a significant decrease in graft loss due to BOS (*p* = 0.049)^31^. A prospective, randomized, open-label, multicenter study of primary lung transplant patients comparing MMF versus azathioprine, found no difference in acute rejection, BOS, or survival between the two groups at 3 years. However, more patients discontinued azathioprine (59.6% vs. 46.5%, *p* = 0.02)^32^. In a randomized, prospective, multicenter trial of 81 lung transplant recipients receiving cyclosporine, corticosteroids, and either MMF or azathioprine, the incidence of biopsy proven grade 2 or greater acute rejection and survival at 6 months did not differ between groups^33^.

Mycophenolate as compared to azathioprine may offer benefit in patients with existing BOS. In a small study of thirteen lung transplant patients with BOS, MMF 1.5 g twice daily was started in place of azathioprine, with resulting stabilization of pulmonary function tests up to 12 months^34^. In clinical practice, patients maintained on azathioprine that develop acute rejection or BOS are frequently switched to mycophenolate.

Mycophenolate is available in two formulations; MMF and an enteric-coated, delayed-release product mycophenolate sodium. The enteric coating on mycophenolate sodium allows for mycophenolic acid to be released directly into the small intestine for absorption rather than in the stomach. MMF is available as an intravenous solution, oral capsules, tablets, and an oral suspension. Conversion between MMF formulations is done at a 1:1 ratio (mg:mg). As opposed to other immunosuppressants, mycophenolate dosing in adults is not based on patient weight, rather is a fixed oral dose. At our center, all lung transplant patients are initially started on MMF 1,000 mg twice daily. The MMF dose can be increased to 1,500 mg twice daily in the setting of rejection and based on tolerability. The MMF dose is generally decreased or held in the setting of severe infections as well as for neutropenia (Figure 2). Routine therapeutic drug monitoring with mycophenolate trough levels is not recommended as there is a poor correlation with drug exposure as measured by the area under the curve^35,36^. However, certain populations are more likely to require individualization of mycophenolate dosing.

Patients maintained on cyclosporine as compared to tacrolimus and patients with cystic fibrosis experience lower mycophenolate exposure and thus may require higher mycophenolate dosing^37,38^. Mycophenolate sodium is only available as enteric coated, delayed-release tablets. Therefore, patients maintained on mycophenolate sodium who cannot take medications orally require conversion to MMF to be given intravenously or as the oral suspension through a feeding tube. Mycophenolate sodium 720 mg is equivalent to 1,000 mg of MMF so this must be considered when switching between products. Mycophenolate is frequently associated with both hematologic (leukopenia, neutropenia) and gastrointestinal (diarrhea, nausea, abdominal pain) adverse effects which may warrant dose adjustment or alternative therapy. In patients maintained on MMF with gastrointestinal adverse effects, switching to mycophenolate sodium may offer benefit^39^. Some medications can lower mycophenolate exposure. Antacids and magnesium salts can decrease the absorption of mycophenolate, therefore separating administration times by at least 2 h can allow for avoiding this interaction. Bile acid sequestrants including cholestyramine should be avoided since they interrupt enterohepatic recirculation and may result in decreased mycophenolate concentrations^40^.

Azathioprine is available as an intravenous solution and an oral tablet with conversion between formulations done at a 1:1 ratio (mg:mg). Azathioprine can also result in leukopenia/neutropenia. Of note, patients with absent or low thiopurine methyltransferase (TPMT) activity are at increased risk of azathioprine associated myelosuppression^41^. Thrombocytopenia, hepatotoxicity, and pancreatitis are also possible adverse effects. Patients on concomitant therapy with medications that inhibit TPMT (i.e., 5-aminosalicylic acid derivatives and furosemide) or xanthine oxidase inhibitors (i.e., allopurinol) may be more susceptible to myelosuppression^42,43^. Azathioprine has a significant drug-drug interaction with allopurinol that results in an increase in the serum concentrations of the active metabolites^44^. In patients that must be maintained on concomitant allopurinol and azathioprine, the azathioprine dose should be reduced to one-fourth of the usual dose and monitored closely for toxicity. Another drug interaction of note is with ribavirin which can be utilized in lung transplant for treatment of respiratory syncytial virus, parainfluenza, and metapneumovirus. Ribavirin may increase serum concentrations of the active methylated metabolites of azathioprine thus when these medications are concomitantly given, increased monitoring for myelosuppression is recommended^45^. At our center, azathioprine is generally utilized second-line if intolerant to mycophenolate (primarily in the setting of gastrointestinal adverse effects) (Figure 2).

### mTOR inhibitors/proliferation signal inhibitors: Sirolimus and everolimus

mTOR inhibitors, also known as proliferation signal inhibitors, including sirolimus and everolimus are utilized for various reasons including as a cell cycle inhibitor alternative in the setting of adverse effects or cytomegalovirus infection, to minimize CNI dosing, as an adjunct agent in the setting of rejection, or in patients that develop or are at high risk for malignancies including post-transplant skin cancers^46^. Despite potential benefits, use of these agents is limited by high rates of adverse events leading to early discontinuation in up to two-thirds of lung transplant patients^47,48^.

Reported use remains relatively infrequent in lung transplantation, with less than 10% of patients maintained on these agents at 1 year post-transplant^1,3^. In a multicenter, randomized, controlled trial of 181 lung transplant patients comparing sirolimus with azathioprine in a tacrolimus-based immunosuppressive regimen, there was no significant difference in the incidence of acute rejection or graft survival between groups although cytomegalovirus infection was less in sirolimus treated patients. Notably, more patients on sirolimus experienced adverse events leading to early discontinuation (64% vs 49%)^47^.

Patients receiving sirolimus also experienced a significantly higher incidence of venous thromboembolism [17.2% vs 3.2%, *p* < 0.01)^49^. In a randomized, double-blind clinical trial of 213 BOS-free lung or heart-lung transplant patients that received everolimus or azathioprine in combination with cyclosporine and corticosteroids, incidence of efficacy failure defined as decline in FEV1 > 15%, graft loss, death or lost to follow-up at 12 months was significantly lower in the everolimus group (21.8% vs. 33.9%, *p* = 0.046); however, at 24 months rates of efficacy failure were similar (43.6% vs 44.6%, *p* = 0.874). Notably, biopsy proven acute rejection at 12 and 24 months was significantly less in the everolimus group: 10.9% vs 25.9%; *p* < 0.001 and 19.8% vs 33.9%; *p* = 0.018, respectively. At 24 months, adverse events were significantly higher in the everolimus group (41.6% vs 19.6%, *p* < 0.01) and 61.4% of patients had discontinued everolimus. Bacterial infections, fungal infections, pneumonia, thrombocytopenia, anemia, and hyperlipidemia occurred significantly more with everolimus^48^. Glanville AR et al. conducted a multicenter, prospective, randomized study of cyclosporine, corticosteroids, and either de novo mycophenolate sodium or delayed-onset everolimus. Three year freedom from BOS Grade 1 and survival was not different between groups based on intention-to-treat analysis but 3-year freedom from BOS Grade 1 was significantly lower in the EVL group per-protocol population. Biopsy proven rejection (*p* = 0.02), leukopenia (*p* < 0.01), diarrhea (*p* < 0.01), and cytomegalovirus infection (*p* = 0.04) were observed more in the mycophenolate group. Venous thromboembolism was more frequent with everolimus (*p* = 0.02)^50^.

Studies examining the impact of mTOR inhibitor on renal function in lung transplant patients have demonstrated inconsistent results. In a small study of sixteen lung transplant recipients 15-96 months post-transplant with renal dysfunction comparing standard CNI based immunosuppression with low-dose CNI based therapy (goal cyclosporine trough 80-120 ng/ml or tacrolimus trough 4-8 ng/ml) plus sirolimus (goal trough 4-8 ng/ml), creatinine clearance improved at 18 months follow-up in the sirolimus group (42.6 mL/min vs. 32.5 mL/min, *p* = 0.05), whereas the control group showed a significant reduction (32.3 mL/min vs. 40.3 mL/min, *p* = 0.02)^51^.

The multi-center NOCTET trial included 282 heart or lung transplant patients (92 lung transplant patients) with renal dysfunction greater than 1 year post-transplant randomized to continue CNI-based immunosuppression or start everolimus with reduced CNI. In all patients, mean change in measured glomerular filtration rate (GFR) from baseline to month 12, was 4.6 mL/min with everolimus and -0.5 mL/min in controls (*p* < 0.0001). However, when examining only lung transplant patients, mean change in measured GFR from baseline to month 12, was 2.3 mL/min with everolimus and -1.3 mL/min in controls (*p* = 0.07). Notably, high rates of adverse effects were reported in the study with edema (29.3% vs. 8.5%, *p* < 0.001), diarrhea (17.1% vs. 5.6%, *p* = 0.003), and leukopenia (11.4% vs. 0%; *p* < 0.001) more frequent with everolimus^52^.

The majority of patients in this study continued their cell cycle inhibitor therefore this could have contributed to additive adverse effects including diarrhea and leukopenia. Importantly, at last follow-up ≥5 years post-randomization, lung transplant patients showed no between-group difference and a similar decline in mean GFR. Pneumonia also was reported to occur more in everolimus treated lung transplant patients versus controls (33.3% vs 9.5%)^53^. In addition to high rates of pneumonia in clinical studies, interstitial pneumonitis is an infrequent but potentially fatal complication of mTOR inhibitors and requires prompt discontinuation of therapy^54,55^.

In terms of concomitant immunosuppression, according to the ISHLT registry data, 5% at one year and 6.1% at five years were maintained on quadruple immunosuppression therapy with an mTOR inhibitor, a CNI, a cell cycle inhibitor and prednisone. At one year and five years follow-up, 1.2% and 6% were maintained on triple immunosuppression therapy with an mTOR inhibitor, CNI, and prednisone whereas only 0.4% at one year and 3% at 5 years were maintained on a CNI free regimen with an mTOR inhibitor, cell cycle inhibitor, and prednisone^3^. CNI free regimens are associated with higher acute rejection rates and in general are not recommended for use in lung transplantation^56,57^. At our center, everolimus or sirolimus are typically reserved for use as an alternate agent in various clinical scenarios (Figure 2). Sirolimus is available both as a tablet and oral solution that could be given feeding tube if needed whereas everolimus is only available in tablet form therefore is not suitable for patients unable to take medications orally. mTOR inhibitor bioavailability may be reduced in lung transplant patients with cystic fibrosis therefore higher doses may be needed to maintain therapeutic levels^57^.

### Co-stimulation blocker: Belatacept

Belatacept, a selective T-cell costimulation blocker, is a CNI alternative approved for use in adult kidney transplantation in combination with basiliximab induction, mycophenolate mofetil, and corticosteroids. It blocks CD28 mediated co-stimulation of T lymphocytes by binding to CD80 and CD86 on antigen-presenting cells, inhibiting T lymphocyte activation and proliferation^58^. Belatacept differs from other available maintenance immunosuppressive agents in terms of route of administration, since it can only be given intravenously. In kidney transplant trials, the rate of PTLD was nine-fold higher in Epstein-Barr virus (EBV) seronegative patients or with unknown serostatus and due to this, belatacept is contraindicated for use in such patients^59^. In clinical trials there were also two fatal cases of progressive multifocal leukoencephalopathy (PML)^59^.

Only small case series have been published on the off-label use of belatacept in lung transplantation mainly for CNI intolerance. Timofte, et al. reported a case series of eight lung transplant recipients with renal insufficiency treated with belatacept while CNI or mTOR treatment was either temporarily discontinued or reduced. Over 6 months, FEV1 remained stable in seven patients. One patient was diagnosed with mild acute cellular rejection (A1). GFR remained stable in two patients and increased in five^60^. Ensor, et al. conducted a single center, retrospective review of eight adult lung transplant recipients that were converted to belatacept after CNI intolerance or failure. Acute cellular rejection, incidence of infection, and FEV1 was not significantly different before and after belatacept although three patients died after conversion^61^. No cases of PTLD were reported in these case series. There was a case report of late invasive tracheobronchial aspergillosis after belatacept use in a lung transplant patient which highlights the need for close monitoring of infection^62^. Overall, more data are needed to further evaluate the safety profile of belatacept and associated risks in lung transplantation.

### Additional considerations in personalizing immunosuppression in lung transplant recipients

Several available immunosuppressive agents including tacrolimus, cyclosporine, sirolimus, and everolimus have a narrow therapeutic index due to significant inter-patient and intra-patient variability. This variability can be due to genetic polymorphisms of cytochrome P450 3A enzymes and the transport protein P-gycoprotein, age, clinical status (time after transplant, liver function, gut function), disease states (i.e., cystic fibrosis), and presence of interacting medications.

While there are several potential pharmacodynamic and pharmacokinetic drug interactions with immunosuppressive agents, those with drug metabolizing enzymes (i.e., CYP 450 enzymes) and drug transporter systems (i.e., p-glycoprotein) are frequently encountered (Table 2). Tacrolimus, cyclosporine, sirolimus, and everolimus are CYP 3A substrates; thus if a medication is instituted that inhibits CYP 3A, increased blood concentrations of the immunosuppressive agent can result. Conversely, medications that are inducers of CYP 3A can lead to decreased blood concentrations of these immunosuppressive agents. The interaction severity can be variable and it is important to consult recommendations for each individual interaction in question for optimal management. Lung transplant patients are frequently maintained on anti-infective agents that interact with immunosuppressive therapy.

Aspergillus is a common fungal infection encountered after lung transplant with many patients receiving systemic azole antifungal agents that are CYP 3A4 and P-glycoprotein inhibitors for prophylaxis and/or treatment. Nontuberculous mycobacterial infections can also be encountered after lung transplant and may require treatment with rifampin or rifabutin which are potent CYP 3A4 inducers^63^. Additionally, over time as survival has improved, transplant patients have an increasing number of co-morbidities such as seizure disorders, HIV, and hepatitis C for which multiple drug-drug interactions often exist. Because of these factors, close therapeutic drug monitoring and associated adjustment of dosing is an important component of post-transplant care. The transplant clinical pharmacist is an essential member of the multi-disciplinary team in assisting with identification and management of such complex drug interactions^64^. Transplant clinical pharmacist involvement with center specific drug therapy guideline development, medication reconciliation and transitions of care, medication therapy management, as well as provider and patient medication education allow for improved safety and decreased medication errors^64,65^.

Age is an important consideration as elderly patients likely require alteration in the immunosuppression regimen due to immunosenescence which is associated with higher rates of diabetes, infection, and malignancy^66^. ISHLT consensus guidelines for the selection of lung transplant candidates state age >65 years is a relative contraindication to transplant in the presence of low physiologic reserve and/or other relative contraindications^67^. Despite this, older patients are increasingly being referred and transplanted. Elderly patients >65 years, comprise over 10% (*n* = 3,789/36,237) of patients in the ISHLT registry data capturing lung transplants from 2004-2015^3^.

There is little published information regarding recommendations for tailoring immunosuppression in the elderly lung transplant patient. A single center retrospective cohort study found patients >65 years experienced no significant difference in incidence of acute rejection or BOS as well as statistically similar 1-year survival after lung transplantation (older group: 79.7% vs younger group: 91.2%, *P* = 0.16). Older and younger patients received maintenance immunosuppression with tacrolimus, mycophenolate mofetil, and steroids although dosing was not reported. However, induction immunosuppression differed in that basiliximab was more commonly utilized for older patients and rATG for younger patients. The predominant reason for death during the first year in the older patients was from infection and authors concluded that immunosuppression protocol adjustments in this population may be warranted^68^. Overall for induction immunosuppression, basiliximab may be preferred in the non-sensitized elderly patient rather than T cell depleting therapies which have been associated with increased risk of infections and malignancy. In terms of maintenance immunosuppression, no significant differences in mycophenolate pharmacokinetics, IMPDH activity or IMPDH inhibition have been demonstrated, although CNI clearance is decreased necessitating lower doses for similar trough levels in elderly kidney transplant recipients^69–71^. Further studies are needed to determine optimal maintenance immunosuppression in elderly patients.

The pharmacogenomics of organ transplantation is an evolving field with several gene polymorphisms under investigation^72,73^. One area of investigation has been regarding the CYP450 3A5 gene polymorphisms. Patients expressing CYP3A5 genotypes need a larger tacrolimus dose requirement than non-expressers to achieve similar trough levels^74,75^. In addition, one of the metabolic pathways of azathioprine involves thiopurine methyltransferase (TPMT). Patients with absent or low TPMT activity are at increased risk of azathioprine associated myelosuppression. TPMT genotyping or phenotyping may assist in identifying patients at risk for developing toxicity and should be considered prior to azathioprine initiation or in patients maintained on azathioprine with myelosuppression unresponsive to dose reduction. Consensus guidelines recommend considering an alternative agent or dose reduction of azathioprine for patients with low or deficient TPMT activity and to start at 30-70% of the target dose for patients with intermediate enzyme activity^41^. Of note, Liang et al. found more acute rejection in heart transplant patients with TPMT genetic variant alleles and concluded such patients should be monitored carefully given this^76^. Although not all gene expression assays are widely available, in the future clinical pharmacogenomics likely will continue to evolve in becoming a routine part of transplant clinical practice.

Infection is common after lung transplant and is the most common cause of death in this population^1^. An immune function assay, Cylex ImmuKnow (ng/mL ATP), has been utilized as a monitoring tool in lung transplant patients with lower levels (≤ 119 ATP ng/ml) correlating with infection^77,78^. Husain and colleagues examined ImmuKnow levels with specific types of infections and found that median values were significantly lower as compared to stable patients (174.8 ng/mL ATP) for cytomegalovirus disease (49.3 ng/mL ATP), viral infection (70 ng/mL ATP), and bacterial pneumonia (92 ng/mL ATP). Of note, patients with fungal colonization had similar ImmuKnow values (167 ng/mL ATP), but were significantly lower (22.5 vs. 183.5 ng/mL ATP; *P* < 0.0001) in colonized patients that then developed fungal disease within 100 days versus those that did not^79^. This suggests a low ImmuKnow value could be predictive of developing infection. It also appears that ImmuKnow values decline over time post-transplant. When examining surveillance ImmuKnow assays, values peaked between 1 week and 1 month after lung transplant and then had a gradual decline over 1 year^77^. While the recommended frequency of monitoring is unknown, serial measurements may be useful in predicting a patient’s risk for infection and allow for opportunity to adjust immunosuppression and/or perform infection surveillance^78,79^. At our center, ImmuKnow assays are often checked in patients with recurrent and severe infections to help guide immunosuppressive therapy adjustments.

## Conclusion

Immunosuppression management after lung transplantation continues to evolve, with an increasing number of agents available for use in various combinations allowing for more choice and individualization of immunosuppressive therapy. Therapeutic developments have led to improved outcomes including lower acute rejection rates and improved survival. While maximizing efficacy and minimizing toxicity of immunosuppressive therapy continues to be a delicate balancing act, maintenance immunosuppression minimization strategies and targeted immune therapy and monitoring continue to advance the transplant immunosuppression field. However, a one size fits all approach for any immunosuppressive strategy may not be best suited to the individual patient and ultimately patient specific factors must be considered when designing the immunosuppressive regimen.

## Competing interests

None

## LIST OF ABBREVIATIONS

CNI: Calcineurin inhibitor
rATG: Rabbit anti-thymocyte globulin
ISHLT: International society for heart and lung transplantation
IL-2 RA: Interleukin-2 receptor antagonist
BOS: Bronchiolitis obliterans syndrome
UNOS: United network for organ sharing
PTLD: Post-transplant lymphoproliferative disorder
MMF: Mycophenolate mofetil
NF-AT: Nuclear factor of activated T-cells
IMPDH: Inosine monophosphate dehydrogenase
TPMT: Thiopurine methyltransferase
FEV1: Forced expiratory volume in the first second
EVL: Everolimus
GFR: Glomerular filtration rate
mTOR: mammalian target of rapamycin
EBV: Epstein-Barr virus
PML: Progressive multifocal leukoencephalopathy
ATP: Adenosine triphosphate
